# Changes in the Gut Microbiome Following Perioperative Prophylactic Cefazolin Administration in Patients Undergoing Orthopedic Surgery: A Longitudinal Prospective Study

**DOI:** 10.3390/antibiotics15070706

**Published:** 2026-07-21

**Authors:** Dokyun Kim, Woo-Suk Lee, Kyoung Hwa Lee, Min Hyuk Choi, Jun Sung Hong, Yu Jin Park, Jihoon G. Yoon, Kwangjun Lee, Seok Hoon Jeong

**Affiliations:** 1 Department of Laboratory Medicine and Research Institute of Bacterial Resistance, Gangnam Severance Hospital, Yonsei University College of Medicine, 211 Eonju-ro, Gangnam-gu, Seoul 06273, Republic of Korea; kyunsky@yuhs.ac (D.K.);; 2 Department of Orthopedic Surgery, Gangnam Severance Hospital, Yonsei University College of Medicine, Seoul 06273, Republic of Korea; 3 Division of Infectious Disease, Department of Internal Medicine, Yonsei University College of Medicine, Seoul 06273, Republic of Korea; 4 Department of Companion Animal Health and Science, Silla University, Busan 46958, Republic of Korea; 5 Division of Zoonotic and Vector Borne Disease Research, Center for Infectious Diseases Research, Korea National Institute of Health, Korea Disease Control and Prevention Agency, Cheongju 28159, Republic of Korea

**Keywords:** cefazolin, microbiome, resistome, prophylactic

## Abstract

Introduction: Cefazolin is a first-generation cephalosporin with a moderate antimicrobial spectrum and the ability to induce the production of beta-lactamases by bacterial hosts. We investigated the effect of prophylactic cefazolin administration on the gut microbiome in patients undergoing orthopedic surgery. Methods: A total of 42 patients were included in this study, and fecal samples were collected before cefazolin administration, within 3 days after administration, and 1 month after surgery. Shotgun whole-metagenome sequencing was performed with DNA extracted from fecal samples to assess the taxonomic composition and antimicrobial resistance genes (ARGs). Results: Within 3 days after perioperative prophylactic cefazolin administration, both the diversity indices and the Gut Microbiome Health Index were significantly decreased. Furthermore, a decrease in two beneficial anaerobic Gram-positive taxa, *Ruminococcus* and *Fusicatenibacter*, and an increase in Enterobacterales was observed. The relative abundances of ARGs related to fluoroquinolone and beta-lactam antimicrobials including penicillin, cephalosporin, carbapenem, and monobactam, were also significantly increased. The changes in the taxonomic composition and resistome related to perioperative cefazolin administration partially reverted after one month. Conclusions: Our findings suggest that even perioperative administration of a single-class antimicrobial agent could be related to the decrease of the gut microbiome diversity with potentially unfavorable taxonomic changes and lead to an increase in ARGs.

## 1. Introduction

The gut microbiome is a complex and dynamic ecosystem with several trillion microbial cells and is considered to have a significant impact on human health [1]. The role of the gut microbiome includes metabolic function and bidirectional interactions with the host, which affects the immune system [2]. Dysbiosis refers to a diseased state of the commensal microbial community accompanied by an imbalance in bacterial composition and a reduction in diversity, resulting in metabolic function changes [3]. The gut microbial ecosystem can be disrupted by several factors. Host-derived factors include genetic background, disease status, and dietary habits [4]. Patients with inflammatory bowel disease present a decrease in the relative abundance of Firmicutes and an increase in the abundance of Proteobacteria in the gut microbiota [5], and chronically ill patients present a significant reduction in the abundances of anti-inflammatory bacterial genera, such as *Faecalibacterium*, in the gut microbiome [6]. Additionally, xenobiotics such as food additives and drugs can also disrupt the homeostasis of the gut microbiome [7].

Therapeutic antimicrobial agents with broad-spectrum antibacterial activity have been demonstrated to cause dysbiosis of the gut microbiota. The administration of fluoroquinolones significantly decreased the fecal concentration of *Enterobacteriaceae* and increased the proportion of enterococci [8]. An association between the use of third- or fourth-generation cephalosporins and the fecal colonization of vancomycin-resistant enterococci has also been described [9]. Furthermore, domination by *Enterococcus*, *Streptococcus*, or Proteobacteria among the gut microbiota after the administration of broad-spectrum antimicrobials was significantly associated with poor clinical outcomes in immunocompromised patients [10].

Cefazolin is a first-generation cephalosporin with a moderate antimicrobial spectrum against Gram-positive cocci except enterococci and some Gram-negative bacilli, including *Escherichia coli*, *Proteus*, and *Klebsiella* [11]. Antimicrobial agents are commonly used for the treatment or prophylaxis of wound infections caused by Gram-positive bacteria. Even though cefazolin has a relatively short half-life of approximately 1.8 h in patients with normal renal function [12], it results in a high peak blood concentration due to the ability of the drug to bind with plasma proteins [13], possibly causing changes in the gut microbiome. Due to the antimicrobial spectrum of this agent, the composition of the microbiota and antimicrobial resistance genes (ARGs) in the gut could be affected by its administration [14,15]. However, the impact of antimicrobial administration on the gut microbiota has rarely been evaluated to date.

Here, a longitudinal prospective study was performed to evaluate changes in the gut microbiome, specifically the gut microbiota composition and diversity and relative abundances of ARGs, via whole metagenomic analyses after very short-term perioperative prophylactic administration of cefazolin in patients undergoing orthopedic surgery.

## 2. Methods

### 2.1. Study Design and Specimen Collection

Patients who underwent orthopedic surgery between August 2022 and January 2023 and who were scheduled for prophylactic antimicrobial administration with standard doses of intravenous cefazolin were enrolled in this study. Standard perioperative prophylaxis was defined as three doses of intravenous cefazolin 1g: one within 1 h before incision, followed by two postoperative doses at 8 h intervals. All patients underwent overnight preoperative fasting, had no bowel preparation, and received parenteral NSAIDs after surgery. Among the 54 patients, 12 patients, including seven for inadequate sample collection, four with a prior history of antimicrobial administration within 3 months or additional antimicrobial administration beyond the predefined schedule during the study period, and one who withdrew consent, were excluded, and a total of 42 patients were ultimately included for further evaluation. A questionnaire about lifestyle factors, including smoking, dietary habits, bowel habits, alcohol and caffeine intake, and exercise, was administered. Three doses of cefazolin were administered to the enrolled patients. Fecal specimens were obtained before surgery (i.e., before the administration of cefazolin), within 3 days after surgery, and 1 month after surgery. The interval from baseline (before surgery) to post-administration (within 3 days after surgery) is defined as the ‘administration phase’, while the period from post-administration to 1 month after surgery is defined as the ‘recovery phase’. The fecal samples were collected in sterile tubes (Para-Pak^®^ Clean vial; Meridian Bioscience, Cincinnati, OH, USA) and were transported to the laboratory maintaining a cold chain at 4 °C, before being stored in a −80 °C deep freezer prior to further processing.

### 2.2. DNA Extraction and Sequencing

Frozen fecal samples were thawed, and bacterial genomic DNA was extracted via the MagMAX^TM^ Microbiome Ultra Nucleic Acid Isolation Kit (Thermo Fisher Scientific Inc., Waltham, MA, USA). Whole metagenome sequencing of the extracted DNA was performed via the Illumina NovaSeq platform (Illumina, San Diego, CA, USA). ZymoBIOMICS microbial community DNA standards (Zymo Research, Irvine, CA, USA) and sterile water were sequenced in every run as positive and negative controls, respectively.

### 2.3. Metagenomic Analysis

Read quality assessment and adapter sequences trimming were performed using fastp (version 1.3.6). Host reads were removed by mapping to the human genome (GRCh38) with Bowtie2 (version 2.5.5) [16]. Taxonomic profiling was performed via Metaphlan version 4.0.6, which compares raw metagenomic reads to the species-specific gene marker database via Bowtie 2 [16]. In addition, raw reads were assembled into contigs via MEGAHIT (version 1.2.9) [17], and contigs shorter than 1 kb were discarded during assembly. Gene predictions were performed via Prodigal (version 2.6.3) [18]. Among the genes predicted, ARGs were searched via the Resistance Gene Identifier (version 6.0.8) and compared with the Comprehensive Antibiotic Resistance Database (version 4.0.1) [19]. The relative abundance of ARGs was expressed as transcripts per million (TPM). Host-removed reads were mapped to the predicted gene catalog with Bowtie2, using default parameters (end-to-end mode), and per gene mapped-read counts were obtained with samtools idxstats (version 1.23.1). A read mapping to more than one gene was assigned to a single best-scoring gene (ties assigned at random). Each gene’s read count was normalized by gene length and then by the per-sample sum of the length-normalized values across all predicted genes. The correlation between the relative abundance of ARGs and the taxonomic composition was calculated by repeated measures correlation [20]. For the mobile genetic element (MGE) analyses, integrons, insertion sequences, and plasmids were detected with IntegronFinder (version 2.0.6), ISEScan (version 1.7.3) and Mob-suite (version 3.1.9). Relative abundance of MGE-associated ORFs were calculated as TPM, same equation with ARG calculation.

### 2.4. Statistical Analysis

Three common diversity indices, the Shannon, Simpson, and Pielou indices and the Gut Microbiome Health Index (GMHI), as suggested by Gupta et al., were calculated [21], and difference in indices among different phases were evaluated by paired Wilcoxon signed-rank test and Friedman test. To control the false discovery rate from testing many taxa and resistance genes, *p* values were adjusted within each analysis using the Benjamini–Hochberg procedure. Principal coordinate analysis (PCoA) was performed to identify differences in taxonomic composition between the groups via Bray–Curtis distance metrics, and the significance between the groups was assessed through permutational multivariate analysis of variance (PERMANOVA), and permutations were restricted within each patient. The statistical significance of the difference among the groups was determined as *p* value < 0.05. Significant taxa for differences between the groups were screened, and their effect sizes were calculated via linear discriminant analysis. A linear discriminant analysis (LDA) score of more than 4.0 with a *p* value under 0.05 was considered to indicate significance. All the statistical analyses were performed via R statistics, and packages including ‘MicrobiotaProcess’ (version 1.14.1), ‘phyloseq’ (version 1.46.0), ‘ggplot2’ (version 3.5.2), ‘tidyverse’ (version 2.0.0), ‘vegan’ (version 2.6.8), ‘coin’ (version 1.4.3), ‘reshape2’ (version 1.4.4), and ‘ggnewscale’ (version 0.5.2) were used for analysis and visualization.

## 3. Results

### 3.1. Clinical Characteristics and the Baseline Taxonomic Distribution and ARG Abundance in the Gut Microbiome

**Figure 1:**
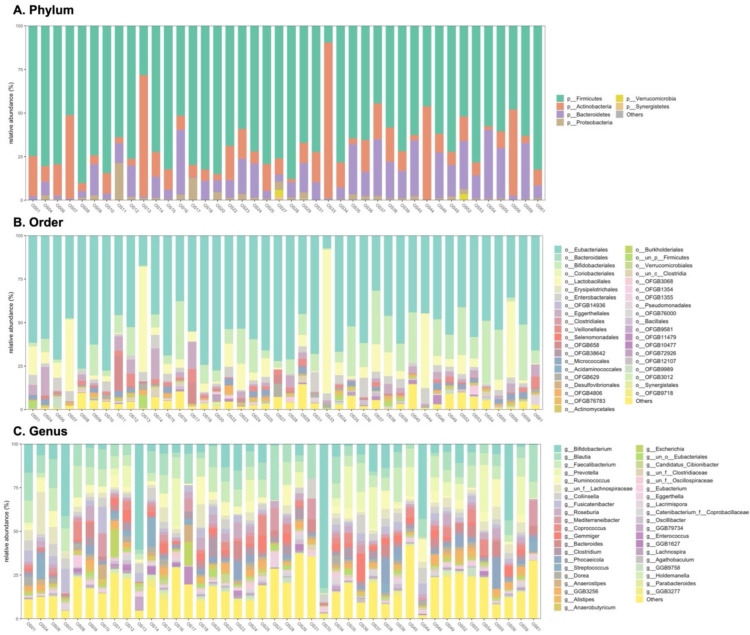
Taxonomic distribution of baseline gut microbiome.

**Figure 2:**
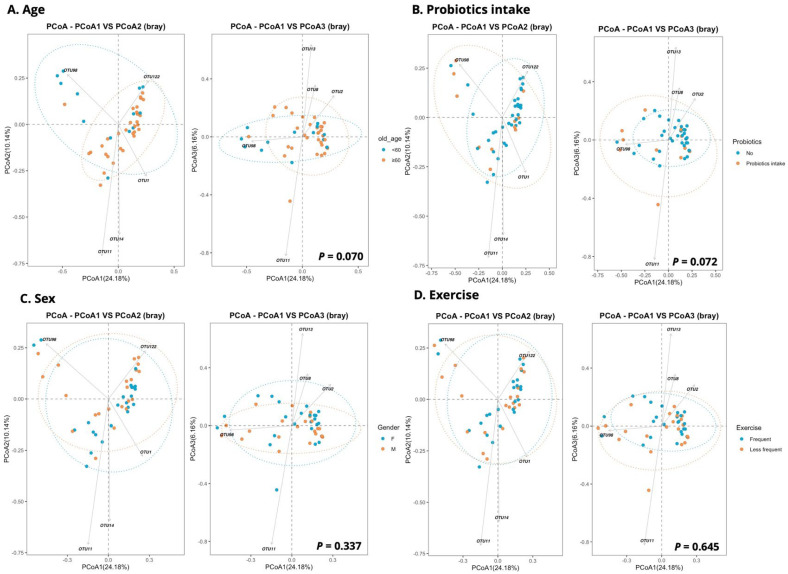
Principal coordinate analysis of the baseline gut microbiome according to the clinical characteristics: (**A**) age; (**B**) probiotics intake; (**C**); sex and (**D**) exercise. Dashed ellipses represent the 95% confidence regions for each group, colored according to the corresponding group.

The median age of the patients was 67.0 years [interquartile range (IQR), 53.0–72.0], and more than half of the patients were female (23/42, 54.8%). The results of the questionnaire about the diet and lifestyle habits of the participants, including smoking, diet, and exercise, are summarized in Table 1. The most common comorbid disease was chronic kidney disease (n = 5, 11.9%), followed by diabetes mellitus (n = 6, 14.3%) and hypertension (n = 4, 9.5%). About half (n = 20, 47.6%) of the patients were overweight (BMI ≥ 25 kg/m^2^). The baseline taxonomic composition of the gut microbiota of the participants is displayed in Figure 1. The most abundant phylum of the baseline gut microbiome was Firmicutes (median value, 70.9%; 1st to 3rd IQR, 61.7–79.6), followed by Actinobacteria (median value, 13.9%; 1st to 3rd IQR, 8.7–23.4), Bacteroidetes (median value, 7.7%; 1st to 3rd IQR, 2.9–14.3), and Proteobacteria (median value, 1.1%; 1st to 3rd IQR, 0.5–2.3) (Figure 1). The baseline gut microbiome was not significantly different according to the patients characteristics such as age (Figure 2A), the sex (Figure 2B), and lifestyle habits including probiotics intake (Figure 2C), and regular exercise (Figure 2D).

A total of 297 ARGs categorized into 29 classes were identified in the gut microbiome of the participants. The ARGs detected in this study are summarized in Table S1. The median relative abundance of all ARGs among the predicted genes before cefazolin administration was 1583.2 TPM (1st to 3rd interquartile range, 1432.6–1828.8). Among the 29 ARG classes, the ARGs against glycopeptides were the most abundant group (median TPM, 845.5), followed by those associated with tetracycline (median TPM, 307.4), those associated with rifamycin (median TPM, 160.1), and those associated with lincosamide (median TPM, 84.8). Most of the ARGs related to glycopeptides were homologous with regulatory genes such as *vanR* of the *van* gene cluster, and *vanA* or *vanB* were rarely identified. The median relative abundance of ARGs encoding cephalosporin and penicillin beta-lactam were 21.4 TPM and 4.2 TPM, respectively. The 73 genes related to cephalosporin resistance were identified in the baseline gut microbiome, and the most frequently identified gene was *bla*_CfxA_ (median TPM, 4.7), followed by *bla*_TEM_ (median TPM, 3.3), H. influenzae PBP3 (median TPM, 3.1), and Acr-related genes (median TPM, 2.2).

### 3.2. Changes in the Taxonomic Composition of the Gut Microbiome After Cefazolin Administration

**Figure 3:**
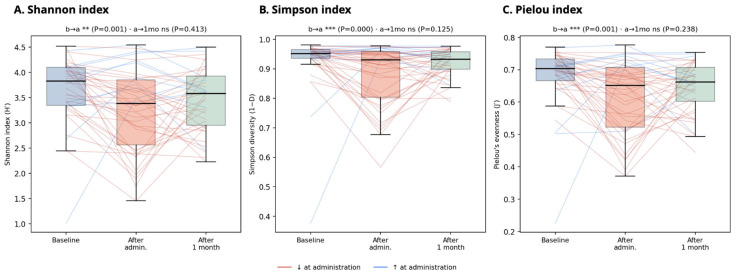
Alpha diversity according to the time period from antimicrobial administration. Box colors indicate the sampling time points: blue, baseline; orange, after administration; green, after 1 month. Differences between consecutive time periods were assessed using the Wilcoxon signed-rank test; asterisks denote the level of statistical significance (**, *p* < 0.01; ***, *p* < 0.001) and ns denotes not significant (*p* ≥ 0.05).

**Figure 4:**
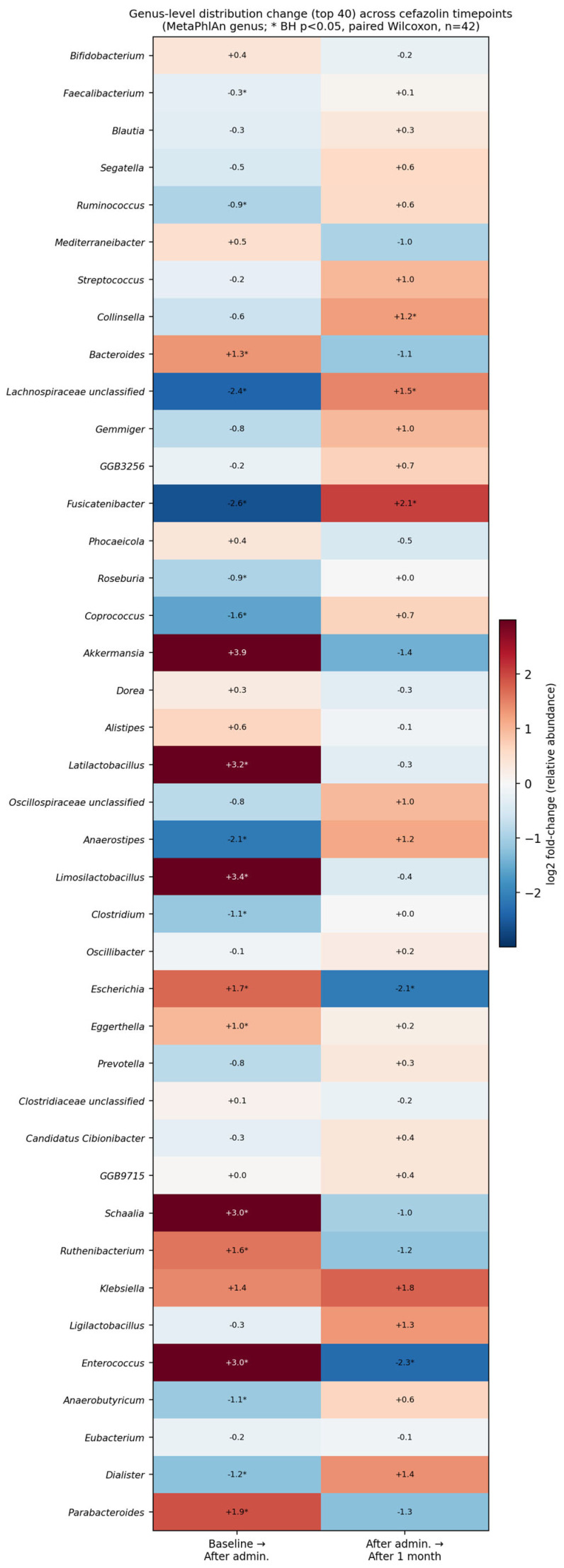
Differences in the relative taxonomic abundances of the 40 most abundant taxa in the administration and recovery phases. The asterisk (*) indicates *p* < 0.05, and *p* values were adjusted within each analysis using the Benjamini–Hochberg procedure.

**Figure 5:**
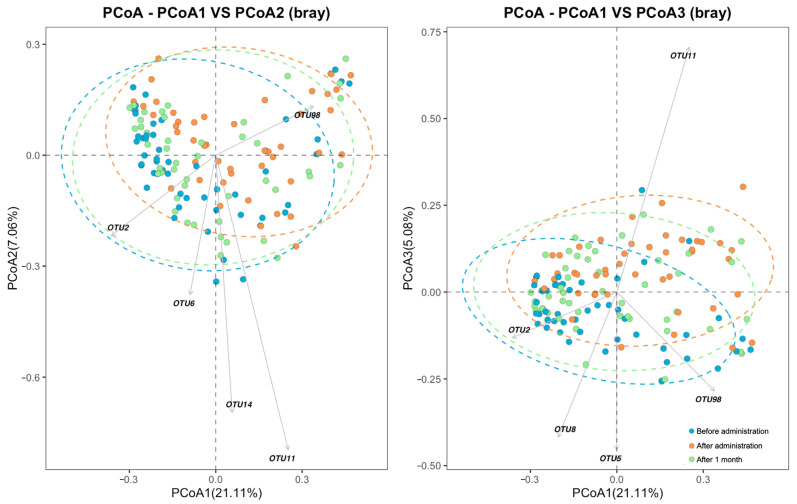
Principal coordinate analysis of the taxonomic component of the gut microbiome according to the time period. Dashed ellipses represent the 95% confidence regions for each group, colored according to the corresponding group.

**Figure 6:**
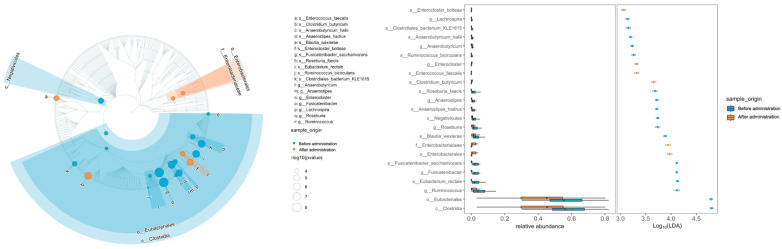
Linear discrimination analysis of the effect size of the gut microbiome before and after antimicrobial administration.

The Shannon diversity of the gut microbiome within 3 days after the administration of cefazolin (median value, 3.38; 1st to 3rd IQR, 2.56–3.85; *p* = 0.001) was significantly lower than that of the baseline gut microbiome (median value, 3.83; 1st to 3rd IQR, 3.35–4.10), similar to the Simpson and Pielou indices (Figure 3). A significant decrease in the median GMHI of the gut microbiome from 0.568 (1st to 3rd IQR, −1.815–1.297) at baseline to −0.138 (1st to 3rd IQR, −3.165–−0.178) was also identified after the administration of cefazolin. Among the 40 most abundant genera in the baseline gut microbiome, 10 genera, including *Fusicatenibacter*, *Faecalibacterium*, *Roseburia*, and *Ruminococcus*, significantly decreased in relative abundance after antimicrobial administration, whereas nine genera, including *Bacteroides*, *Latilactobacillus*, *Escherichia*, *Schaalia*, and *Enterococcus*, significantly increased in relative abundance (Figure 4). PCoAs also revealed a significant difference between the bacterial taxonomic components before and after prophylactic antimicrobial administration (PERMANOVA *p* value = 0.0004); *Faecalibacterium prausnitzii* (OTU2), *Fusicatenibacter saccharivorans* (OTU6), *Bifidobacterium longum* (OTU11), *Bifidobacterium pseudocatenulatum* (OTU14), and *Ruminococcus gnavus* (OTU88) were the taxa responsible for the difference (Figure 5). LDA showed opposite changes in proportions between bacterial orders after cefazolin administration: an increased concentration of Enterobacterales but a decreased concentration of *Eubacteriales* (Figure 6). The genera, *Ruminococcus* and *Fusicatenibacter*, which are in the order *Eubacteriales*, were significantly decreased with high LDA scores; whereas, no specific genus was significantly increased in members of the order Enterobacterales.

The changes in the taxonomic composition of the gut microbiome after one month generally showed opposite trends compared with those within 3 days after antimicrobial administration at the genus level. Among the 40 abundant genera, the relative abundances of three genera, including *Collinsella*, *Lachnospiraceae* unclassified, and *Fusicatenibacter*, were significantly increased; whereas, that of *Enterococcus,* were significantly decreased (Figure 4). The PCoAs also revealed that the taxonomic composition of the gut microbiota after one month was similar to that at baseline. A contrary aspect of the GMHI recovery after one month was identified. Among the 38 of 42 patients whose GMHI decreased after antimicrobial administration, 27 (71.1%) recovered by one month, whereas 11 (28.9%) showed a further decrease. No clinical variable was significantly associated with the failure of GMHI recovery.

### 3.3. Changes in the Relative Abundance of ARGs in the Gut Microbiome

**Figure 7:**
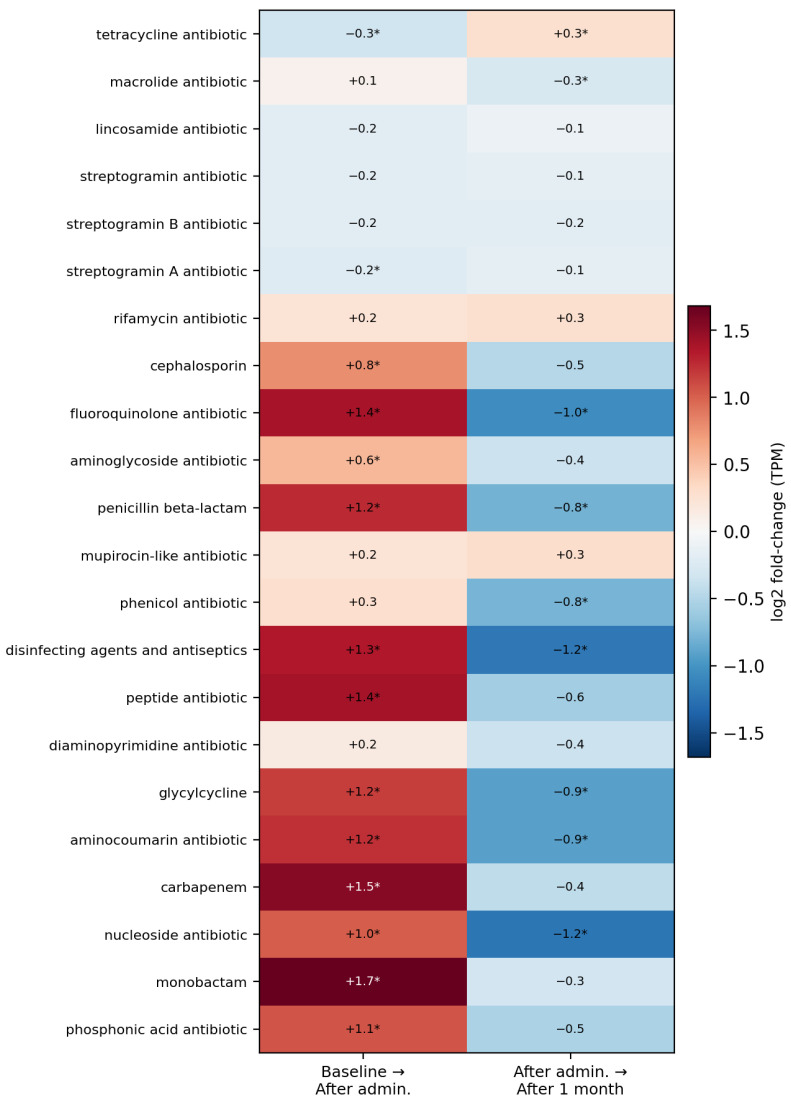
Changes in the abundances of antimicrobial resistance genes in the gut microbiome. The asterisk (*) indicates *p* < 0.05, and *p* values were adjusted within each analysis using the Benjamini–Hochberg procedure.

The total number of ARGs among the predicted genes slightly increased from 1583.2 TPM at baseline to 1695.4 TPM within 3 days after cefazolin administration, but the difference was not statistically significant. By the ARG class, 15 drug classes were significantly enriched after administration (Benjamini–Hochberg-adjusted *p* < 0.05, paired Wilcoxon; Figure 7). The ARGs associated with cephalosporin (from 21.4 to 84.1 TPM; adjusted *p* = 0.004), penicillin beta-lactam (4.2 to 36.8; *p* < 0.05), carbapenem (0 to 8.7; *p* < 0.05) and monobactam (0 to 5.2; *p* < 0.05) were significantly increased after the perioperative cefazolin administration. Resistance to aminoglycosides (18.0 to 39.2; *p* < 0.05), peptide antimicrobials (0 to 15.9; *p* < 0.05), disinfectants and antiseptics (10.0 to 41.9; *p* < 0.05), glycylcycline (2.9 to 20.1; *p* < 0.05) and phenicols (10.7 to 27.2; *p* < 0.05), as well as diaminopyrimidine, phosphonic-acid, nucleoside, aminocoumarin, and elfamycin resistance, were also significantly increased.

One month after the administration of the prophylactic antimicrobial, the median value of the total ARGs was 1554.0 TPM (1st to 3rd IQR, 1264.5–1914.0). All drug-class groups that had been relatively increased during administration reverted in the recovery phase (Figure 7), such as fluoroquinolone (from 92.2 to 23.3 TPM), cephalosporin (from 84.1 to 23.4 TPM), penicillin β-lactam (from 36.8 to 2.7 TPM) and aminoglycoside (from 39.2 to 21.1 TPM). These reversions were not statistically significant after Benjamini–Hochberg correction across all classes.

The abundance of plasmid-borne genes increased after administration and reverted by one month, similar to the resistome dynamics, whereas insertion-sequence abundance was unchanged; plasmid abundance correlated with several ARG classes (Figure S1).

### 3.4. Correlation Between the Taxonomic Composition and Relative Abundances of ARGs in the Gut Microbiome

**Figure 8:**
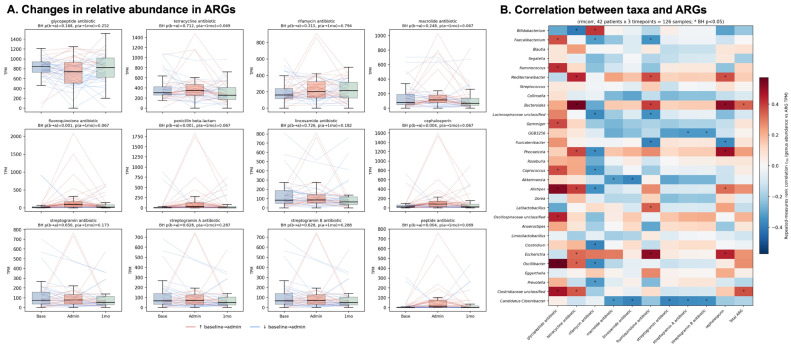
Correlation analysis between the relative taxonomic abundances and antimicrobial resistance genes. The box plots (**A**) represent the abundance of ARGs across three time points including baseline (blue), after cefazolin administration (red), and after one-month since administration (green). The heatmap (**B**) indicates the correlation between taxa and ARGs. Correlation analysis was performed with repeated measures correlation [20]. The asterisk (*) indicates *p* < 0.05, and *p* values were adjusted within each analysis using the Benjamini–Hochberg procedure.

Correlation analyses between the relative abundances of ARGs and the proportion of taxonomic composition were performed to determine the taxa responsible for the increased abundances of ARG classes, and the significant correlations are summarized in Figure 8. Among the top 40 genera, *Escherichia* showed significant correlations with ARG classes related to fluoroquinolone (*p* < 0.05) and cephalosporin antimicrobials (*p* < 0.05), which were the ARG classes enriched after perioperative cefazolin administration. *Mediterraneibacter* and *Bacteroides* were also identified as a major contributor to the resistome profile in cephalosporin and fluoroquinolone antimicrobials. In contrast, *Faecalibacterium*, *Fusicatenibacter*, and *Lachnospiraceae* exhibited negative correlations with these ARG classes.

## 4. Discussion

The effects of fluoroquinolones, 3rd-generation cephalosporins, carbapenems, or tetracyclines on alterations of the gut microbial community have been investigated in previous longitudinal studies comparing the gut microbiota of collected stool samples from recruited healthy people before and after drug administration [22,23,24]. However, the study models have potential problems in terms of ethical issues and difficulty recruiting healthy volunteers. Scheduled antimicrobial administration, such as prophylaxis of surgical site infection before clean surgeries, could be an alternative study model without the recruitment of healthy volunteers or ethical issues. Orthopedic surgeries are generally performed on individuals with few medical problems, except simple fractures or degenerative joint disease. Indeed, the baseline gut microbiota of the patients in this study showed a high proportion of Firmicutes and a relatively low prevalence of Bacteroidetes and Proteobacteria at the phylum level, similar to previous reports of healthy individuals in South Korea [25]. Additionally, in the genus-level analyses, the top five most abundant genera, namely, Faecalibacterium, Bifidobacterium, Blautia, Ruminococcus, and Collinsella, were reported to be prevalent in the gut microbiota of healthy individuals. These findings suggest that our study design could be an alternative model for evaluating the impact of antimicrobial use on the gut microbiome without the recruitment of healthy individuals.

In our study, the alpha diversity including Shannon, Simpson, and Pielou indices and the GMHI of the gut microbiota were significantly decreased within 3 days after prophylactic cefazolin administration, consistent with the previous report with cefazolin administration in the patients underwent spinal surgery (Shannon index, 4.49 to 4.43; Simpson index, 0.92 to 0.87) [26]. The GMHI was developed as a health-status index rather than a measure of short-term antibiotic perturbation; accordingly, the decreased GMHI observed here is interpreted as an unfavorable compositional shift rather than a clinical health assessment. The opposite trend of changes in the relative abundance of bacterial genera among the gut microbiota, i.e., decreases in two beneficial anaerobic Gram-positive taxa, *Ruminococcus* and *Fusicatenibacter,* but increases in *Enterococcus*, after the administration of cefazolin was consistent with the expected compositional changes in the gut microbial taxa on the basis of the antimicrobial spectrum of cefazolin. However, unexpected changes in gut microbial taxa after drug administration and increases in the relative abundances of Enterobacterales and *Bacteroides* were also identified [10,27,28,29]. These changes might have resulted from acquired beta-lactam resistance determinants in *Escherichia* and *Klebsiella*, which are the most common genera in Enterobacterales, considering the significant correlations between those taxa and ARGs related to cephalosporins. In addition, the aggregate relative abundance of recognized 11 butyrate producers including *Faecalibacterium*, *Roseburia*, *Coprococcus*, *Anaerostipes*, *Fusicatenibacter*, *Subdoligranulum*, *Butyrivibrio*, *Anaerobutyricum*, *Agathobaculum*, *Butyricicoccus*, and *Oscillibacter* decreased markedly in the administration phase (median 0.22 to 0.10; paired *p* value < 0.001) and partially recovered by one month [30]. Our findings suggest that even short-term administration of cefazolin could lead to a decrease in gut microbiome diversity with unfavorable compositional changes.

The gut resistome profile can vary according to the country, but high relative abundances of ARGs have been reported in most populations [31], similar to our study. The relative increase in beta-lactamase genes after the administration of cefazolin might be due to an increase in the relative abundance of *Enterobacterales,* including *Escherichia*. Notably, the relative abundances of ARGs related to fluoroquinolone, beta-lactam antimicrobials including penicillin, cephalosporin, carbapenem, and monobactam were also significantly increased, probably due to co-carriage with the beta-lactamase genes in Enterobacterales and the relative increase in the abundance of *Enterococcus* intrinsically harboring PBP point mutations, which results in low-does cephalosporin resistance [32]. In addition, although glycopeptide-class ARGs were the most abundant group at baseline, they mostly consisted of *van* operon homologues associated with accessory or regulatory components, which were detected as low-identity RGI Strict hits, and core resistance determinants rarely identified, Therefore, this finding indicates distant homology to *van*-cluster genes in commensal gut anaerobes rather than functional vancomycin resistance, and should be interpreted with caution rather than as meaningful resistance.

The gut microbiota of the patients showed partial resilience to preantimicrobial conditions one month after antimicrobial administration. The opposite trend was observed within three days after antimicrobial administration: *Bacteroides*, *Mediterraneibacter*, *Phocaeicola*, *Parabacteroides*, and *Escherichia* showed a decrease in relative abundance; whereas, *Faecalibacterium*, *Blautia*, and *Collinsella* exhibited an increase. Several mechanisms, including bacteria–bacteria cooperation, effects on the host immune system, genetic factors, and environmental conditions such as dietary and lifestyle habits, might support the resilience of the gut microbiome [33,34]. Although we could not identify the significant factors related to the failure of the gut microbiome to recover in the present study due to the limited number of enrolled patients, clinical conditions such as age, underlying diseases, baseline community diversity, or the severity of disturbance might be important factors, and dietary change or difference could affect the microbiome resilience, which could not be identified due to observational nature of this study. Further investigation is needed.

Our study has several limitations. First, the study included a small, ethnically homogeneous (single-center Korean) cohort, which limits statistical power and the generalizability of our findings to other ethnic or racial populations. Second, the changes in the gut microbiota were investigated by comparing the relative abundance of each taxon, and the absolute extent of the decrease in bacteria was not identified. Third, although the changes in the gut microbiome observed in this study are consistent with the antimicrobial spectrum of cefazolin, the effects on the gut microbiome during orthopedic surgery, including the use of anesthesia, analgesics, and systemic inflammation, could not be assessed due to the study design. Finally, functional and host biomarkers including intestinal pharmacokinetics, gut permeability, and mucosal inflammation were not assessed. Further investigation about the host response should be performed.

In conclusion, the short-term perioperative administration of a single-class antimicrobial agent, cefazolin, might cause a significant decrease in gut microbial diversity along with potentially unfavorable changes in taxonomic composition, an increase in the relative abundances of ARGs associated with multiple classes of antimicrobials were identified. Caution should be taken with antimicrobial administration to help maintain the homeostatis of the gut microbiome and limit the possible increase in the relative abundance of antimicrobial resistance genes.

## Figures and Tables

**Table 1:** Clinical characteristics.

Variable	Total (n = 42)
Age	67.0 [53.0–72.0]
Female	23 (54.8)
Underlying comorbidities	
Hypertension	4 (9.5)
Diabetes mellitus	6 (14.3)
Liver disease	4 (9.5)
Chronic kidney disease	5 (11.9)
Body mass index	24.5 [22.6–26.7]
Type of surgery	
Total knee arthroplasty	18 (42.9)
Arthroscopic repair	8 (19.0)
Open reduction in fracture	3 (7.1)
Total hip arthroplasty	4 (9.5)
Others	9 (21.4)
Probiotic administration	11 (26.2)
Smoking habits	
Nonsmoker	33 (78.6)
Ex-smoker	7 (16.7)
Smoker	2 (4.8)
Diet habits	
Greasy food	10 (23.8)
Vegetables	26 (61.9)
Fermented food	11 (26.2)
Salted food	23 (54.8)
Regular stool habit	17 (40.5)
Frequent alcohol intake	14 (33.3)
Frequent caffeine intake	31 (73.8)
Regular exercise	11 (26.2)

## Data Availability

The data generated during the current study are available in the National Center for Biotechnology Information Sequence Read Archive under PRJNA1200790.

## Supplementary Materials

The following supporting information can be downloaded at: https://www.mdpi.com/article/10.3390/antibiotics15070706/s1. Table S1. Antimicrobial resistance genes detected in the gut microbiome across the three sampling time points. Figure S1. Mobile genetic elements in the gut microbiome across the three sampling time points. (A) Relative abundance of plasmid-borne genes and insertion-sequence (IS)-associated genes at baseline, after cefazolin administration, and one month after surgery. (B) Spearman correlation coefficient between plasmid- or IS-associated gene abundance and the relative abundance of individual antimicrobial resistance gene classes.
