# Supplementary figures and images for: Changes in the Gut Microbiome Following Perioperative Prophylactic Cefazolin Administration in Patients Undergoing Orthopedic Surgery: A Longitudinal Prospective Study
Source: Antibiotics (Basel). 2026 Jul 21;15(7):706. doi: 10.3390/antibiotics15070706 (PMC13405979; doi:10.3390/antibiotics15070706)

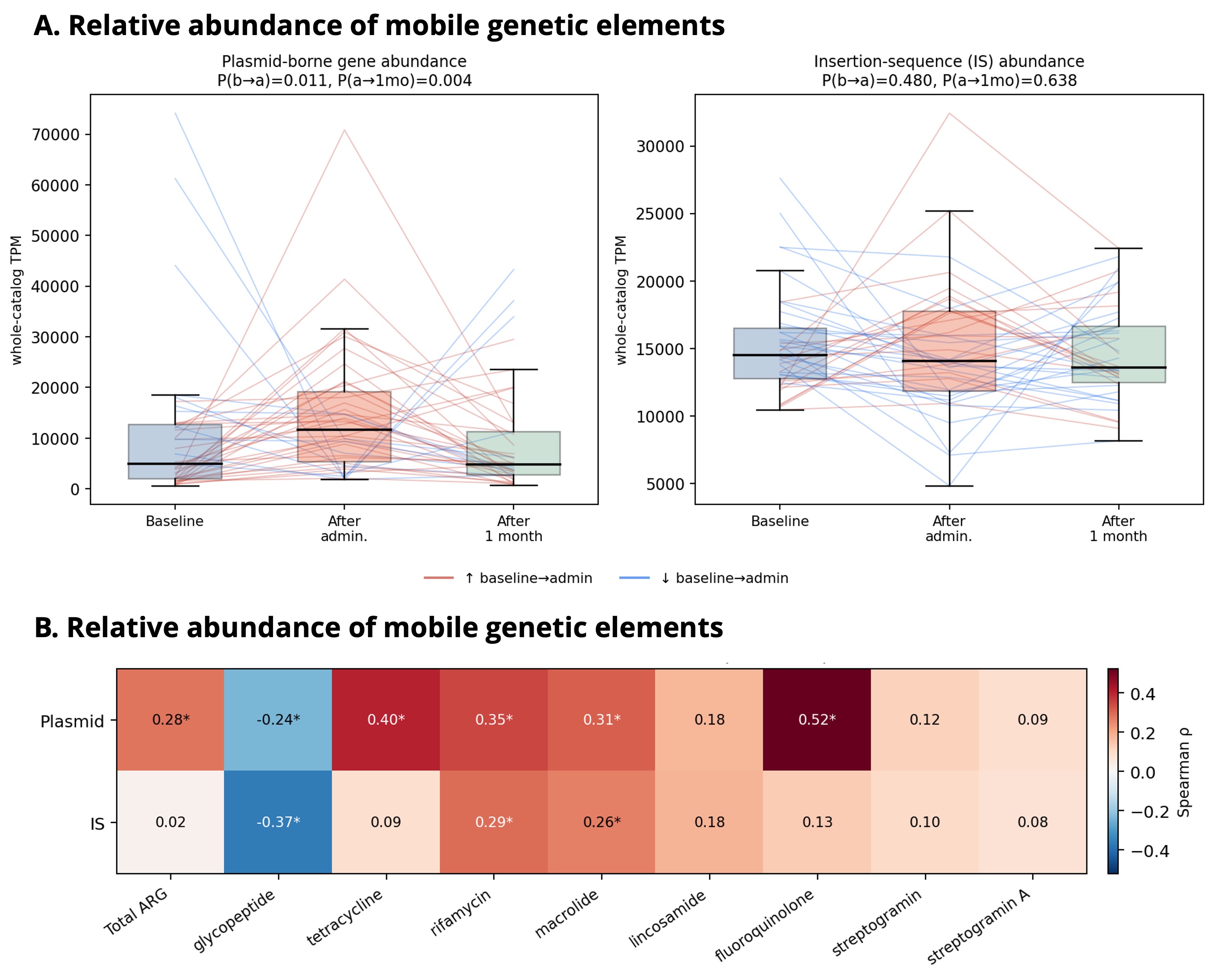

Supplement: Supplementary file 1 [file antibiotics-15-00706-s001.zip › Figure S1.jpg]
